# Corrupted DNA-binding specificity and ectopic transcription underpin dominant neomorphic mutations in KLF/SP transcription factors

**DOI:** 10.1186/s12864-019-5805-z

**Published:** 2019-05-24

**Authors:** Melissa D. Ilsley, Stephen Huang, Graham W. Magor, Michael J. Landsberg, Kevin R. Gillinder, Andrew C. Perkins

**Affiliations:** 1 Mater Research, Translational Research Institute, University of Queensland, Brisbane, QLD Australia; 2 School of Biomedical Sciences, University of Queensland, Brisbane, QLD Australia; 3 School of Chemistry and Molecular Biosciences, University of Queensland, Brisbane, QLD Australia; 4 Australian Centre for Blood Diseases, Monash University, Melbourne, VIC Australia

**Keywords:** KLF1, CDA, 4sU-RNA labeling, Hemolysis, ChIP-seq

## Abstract

**Background:**

Mutations in the transcription factor, *KLF1*, are common within certain populations of the world. Heterozygous missense mutations in *KLF1* mostly lead to benign phenotypes, but a heterozygous mutation in a DNA-binding residue (E325K in human) results in severe Congenital Dyserythropoietic Anemia type IV (CDA IV); i.e. an autosomal-dominant disorder characterized by neonatal hemolysis.

**Results:**

To investigate the biochemical and genetic mechanism of CDA IV, we generated murine erythroid cell lines that harbor tamoxifen-inducible (ER™) versions of wild type and mutant KLF1 on a *Klf1*^−/−^ genetic background. Nuclear translocation of wild type KLF1 results in terminal erythroid differentiation, whereas mutant KLF1 results in hemolysis without differentiation. The E to K variant binds poorly to the canonical 9 bp recognition motif (NGG-GYG-KGG) genome-wide but binds at high affinity to a corrupted motif (NGG-GRG-KGG). We confirmed altered DNA-binding specificity by quantitative in vitro binding assays of recombinant zinc-finger domains. Our results are consistent with previously reported structural data of KLF-DNA interactions. We employed 4sU-RNA-seq to show that a corrupted transcriptome is a direct consequence of aberrant DNA binding.

**Conclusions:**

Since all KLF/SP family proteins bind DNA in an identical fashion, these results are likely to be generally applicable to mutations in all family members. Importantly, they explain how certain mutations in the DNA-binding domain of transcription factors can generate neomorphic functions that result in autosomal dominant disease.

**Electronic supplementary material:**

The online version of this article (10.1186/s12864-019-5805-z) contains supplementary material, which is available to authorized users.

## Background

KLF1, Krüppel-like factor 1, is an erythroid-specific transcription factor (TF) [1] that coordinates almost all aspects of erythropoiesis [2–4]. It is the founding member of a family of 17 TFs which are highly related to the SP1 family. *Klf1*^−/−^ mice die in utero from severe anemia [5, 6] and *KLF1* null humans display severe *hydrops fetalis*, which is lethal without intervention [7]. On the other hand, *Klf1*^+/−^ mice, and humans with heterozygous mutations in *KLF1,* have mild phenotypes. A few KLF1-dependent target genes are sensitive to haplo-insufficiency, so one can find blood group serological abnormalities, such as In(Lu) and elevated HbF and HbA_2_ levels in carriers if one specifically searches for them [7–11]. On the other hand, red blood parameters such as cell size (MCV) are normal, so carriers are difficult to discover via routine full blood examination (FBE). This explains why *KLF1* variants have not been found in genome-wide association studies (GWAS) of variations in the FBE [12], despite mutations occurring at very high frequencies in some populations [13]. In fact, most carriers remain undetected throughout life.

Congenital Dyserythropoietic Anemia type IV (CDA IV) is a rare autosomal dominant erythrocyte disorder (OMIM: 613673) characterized by dyserythropoiesis and hemolysis. Since 2010, six unrelated patients with CDA IV have been identified with the same mutation in KLF1 (c.973G > A; p.E325K) [14–18]. The patients have markedly elevated HbF, nucleated RBCs in the peripheral blood, splenomegaly, and growth delay. They are transfusion dependent from early life [16, 17]. The glutamic acid residue in the second zinc finger (ZF2) of KLF1 (i.e. E325 at + 3 relative to the start of the preceding α-helix) is conserved in all KLFs and SP proteins and plays a structural role in recognition of the central pyrimidine nucleotide on the G-rich strand of the 9 bp DNA recognition sequence (NGG-GYG-KGG) [19].

**Fig. 1:**
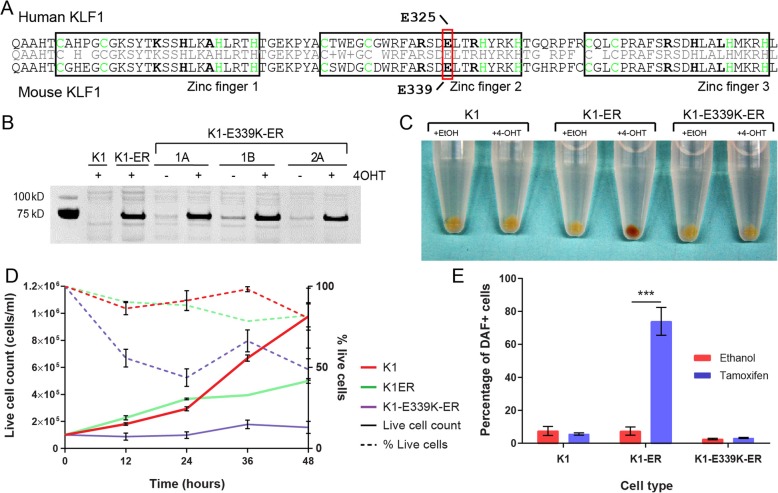
An in vitro cell line model to study human CDA type IV. **a** Alignment of human and mouse KLF1 demonstrating the sequence conservation within the C_2_H_2_ zinc finger domains. Mutations associated with CDA IV (E325K) and *Nan* (E339D) are indicated by boxes. Bold amino acids indicate residues which contact DNA when bound. **b** Western blot of nuclear extracts from cell lines generated in this study. The blot shows presence of KLF1-ER in the nucleus after induction with 4-OHT (+) in a K1-ER cell line and 3 independent clones of the K1-E339K-ER cell line. Full-length blot image is presented in Additional file 1: Figure S1. **c** Cell pellets from K1 (parental), K1-ER (wild type), and K1-E339K-ER (CDA mutation) lines treated with 4-OHT or ethanol (vehicle control) for 48 h. The redness of the pellet indicates production of hemoglobin. **d** Cell number and viability measured at 12-h intervals following induction with 4-OHT for K1 (yellow), K1-ER (green), and K1-E339K-ER (purple) cells. Solid line represents number of live cells in culture and dashed line represents the percentage of live cells in culture. Error bars show ± SEM from three independent clones or three biological replicates for parental K1 cells.**e** Percentage of DAF positive cells (indicating presence of hemoglobin) at 48 h post-treatment with 4-OHT or ethanol. Data are represented as mean ± SEM from 3 clonally independent cell lines. ***P < 0.001 (Student’s t-test)

An ENU mutant mouse strain (the neonatal anemia or *Nan* mouse) harbors a mutation in the equivalent position to human KLF1-E325 (i.e. E339D in mouse) [20–23] (Fig. 1a). Like human CDA IV patients, heterozygous *Klf1*^*+/Nan*^ mice also exhibit neonatal hemolysis. Furthermore, *Klf1*^*Nan/Nan*^ mice die at embryonic day E10–11 due to severe defects in primitive hematopoiesis. This phenotype is more severe than a complete loss of function of *Klf1* [5, 6]. We previously showed the KLF1-E339D protein binds to a degenerate DNA motif in vitro and in vivo, and this corrupts the erythroid transcriptome leading to hemolysis [24–26]. That is, KLF1-E339D has a neomorphic biochemical function which results in red blood cell destruction.

We hypothesized the E325K mutation in human KLF1 has a similar neomorphic DNA-binding function which would result in aberrant gene expression and toxic effects in human erythroid cells. Furthermore, we hypothesized the E > K variant would be more deleterious than the more conservative E > D amino acid change. Due to the rarity of patients, we generated erythroid cell lines carrying an Estrogen Receptor (−ER) fusion of either mutant or wild type KLF1 on a *Klf1*^−/−^ genetic background that could be conditionally activated with 4-hydroxytamoxifen (4-OHT) [27]. The experimental design and cellular context is identical to that which we employed to study the function of KLF1-E339D [24], so we could directly compare the biochemical and genetic functions of the two variants.

Nuclear translocation of murine KLF1-E339K-ER results in rapid erythroid cell destruction without differentiation, whereas induction of wild type KLF1-ER induces controlled terminal differentiation. We employed ChIP-seq to show that KLF1-E339K-ER differentially occupies binding sites genome-wide and exhibited preference for an altered recognition motif in vivo. We confirmed altered DNA-binding specificity of KLF1-E339K compared to wild type KLF1 using purified recombinant zinc finger domains (zf) in quantitative electromobility shift assay (EMSA assays). The observed change in specificity at position 5 on the G-rich strand is consistent with structural studies [19], yet is distinct from that which we previously determined for KLF1-E339D-zf [24]. Ultimately, the altered DNA-binding specificity of KLF1-E339K-zf results in ectopic transcription of ~ 100 non-erythroid genes. Together these genes are likely to derail normal erythroid differentiation and induce hemolysis. Our results provide a molecular understanding of how neomorphic DNA-binding specificities in TFs can result in dominant genetic disease. Given the large number of TFs encoded in the human genome, we propose this novel mechanism will be broadly applicable.

## Results

### An inducible cell line system to study CDA type IV

Congenital Dyserythropoietic Anemia (CDA) type IV (OMIM: 613673) is a severe autosomal dominant disease due to a missense mutation in the DNA-binding domain of human KLF1 (E325K) [17]. We hypothesized this mutation would generate a mutant TF with aberrant DNA-binding specificity*,* and this would induce aberrant gene expression and cell death. To define an in vivo DNA-binding specificity of the E > K variant KLF1 protein we sought to undertake ChIP-seq which was not confounded by the presence of wild type KLF1. This would not be possible with primary patient samples because they have one normal *KLF1* allele. Rather, we required a *KLF1* null erythroid cell system in which we could rescue KLF1 function. To achieve this, we utilized an immortalized murine *Klf1* null cell line, K1 [27]. From these, we created stable transgenic sublines in which KLF1 is retained in the cytoplasm by means of fusion to the ligand-binding domain of the estrogen receptor (ER™) modified to respond only to 4-hydroxytamoxifen (4-OHT). An E to K mutation was introduced in the second zinc finger of murine Klf1 (E339K) to model the human KLF1-E325K variant (Fig. 1a). In this K1-E339K-ER system, addition of 4-OHT to the culture induces rapid nuclear translocation, DNA-binding and activation of gene expression (Fig. 1b and Additional file 1: Figure S1).

We previously generated and characterized *Klf1*^−/−^ cell lines in which wild type KLF1-ER is inducible by 4-OHT; i.e. K1-ER cells (previously known as B1.6 cells) [24, 27]. They express equivalent amounts of KLF1-ER in the nucleus after 4-OHT treatment to K1-E339K-ER cells (Fig. 1b) and K1-E339D-ER cells [24], allowing direct comparison of the biological effects and biochemical functions of wild type, E > K and E > D mutant versions of KLF1-ER in the endogenous erythroid chromatin context. Upon induction with 4-OHT, K1-ER cells slow proliferation and differentiate as can be seen by hemoglobinization of the cell pellet [24, 27] (Fig. 1c-e). In contrast, K1-E339K-ER cells produce no detectable hemoglobin upon addition of 4-OHT, cease proliferating and ultimately die rather than differentiate (Fig. 1d), suggesting a deleterious or toxic effect of KLF1-E339K-ER.

### The E > K mutation results in altered DNA binding specificity in vivo

**Fig. 2:**
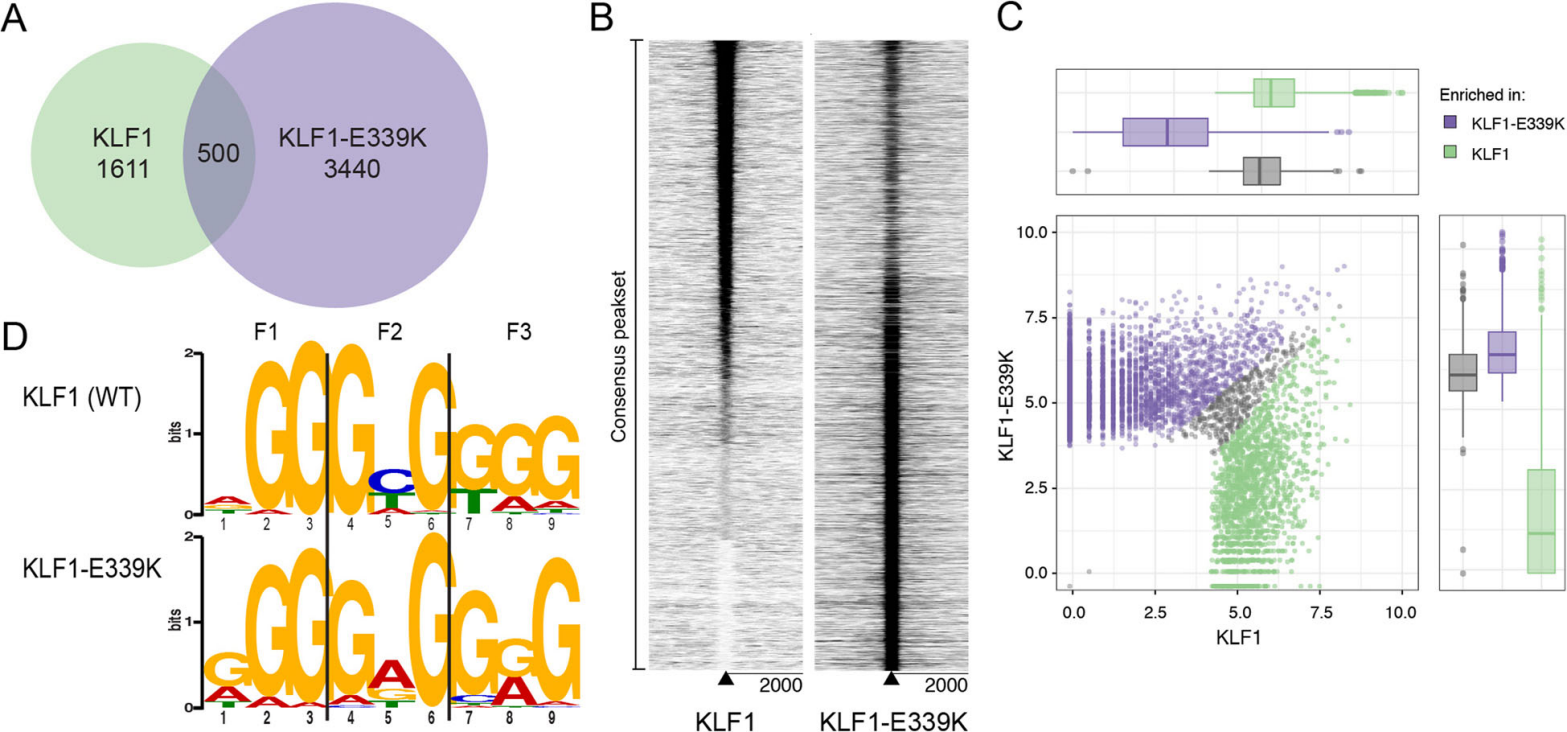
KLF1-E339K-ER binds to novel sites throughout the genome due to a change in DNA-binding specificity. **a** A Consensus occupancy peakset was generated from overlapping KLF1-ER and KLF1-E339K-ER binding sites. **b** Heat maps of read density of KLF1-ER and KLF1-E339K-ER ChIP-seq at a ± 2 kb from the consensus peakset. Plots are clustered according to the nearest neighbor chain algorithm [50]. **c** Scatter plot with marginal boxplots displaying the log_2_ normalized mean read count from differential binding analysis between KLF1 and KLF1-E339K peaksets and read alignments. **d** Position weighted matrix for the highest-ranking motif derived from KLF1-ER (top) and KLF1-E339K-ER (bottom) peaks (±50 bp), as determined by MEME

To assess how the E > K mutation affects DNA-binding of KLF1-ER, we undertook ChIP-seq 3 hours after 4-OHT induction using an antibody to the ligand-binding domain of the estrogen receptor (ERα) [28] (See Methods). Occupied genomic regions were identified using MACS2 [29] and GEM [30] across samples to remove peak caller bias. A consensus peakset of 5551 sites was obtained including only 500 co-occupied and 3440 unique to KLF1-E339K-ER, indicating a large number of novel sites are bound by KLF1-E339K-ER in vivo (Fig. 2a). To observe the change in binding genome-wide, we plotted read density heat maps for each dataset. Figure 2b suggests KLF1-E339K-ER binds at sites in the genome that are not bound by KLF1-ER, but it can also bind weakly to sites normally bound by KLF1-ER. To further analyze differential binding between these factors, we used DiffBind [31] to analyze the consensus peakset for levels of read enrichment (Fig. 2c). We identified 5173/5551 consensus sites that were differentially bound with a false discovery rate of < 0.05 (Additional file 5: Table S1). Together, this suggests both a neomorphic and hypomorphic function for the E > K mutation, just as for the E > D mutation [24]. De novo motif analysis using MEME revealed KLF1-E339K-ER binds to an altered GC-rich motif in vivo compared to wild type KLF1-ER (Fig. 2d). KLF1-E339K-ER binds to the motif NGG GRG KGG (E-value = 7.6 × 10^− 2168^). The major divergence from the wild type KLF1-ER position weighted matrix (PWM) (NGG GYG KGG; E-value = 1.8 × 10^− 1140^) is a change in the fifth nucleotide from a pyrimidine (i.e. Y = C or T), to a purine (i.e. R = A or G) (Fig. 2d). Local motif enrichment analysis using CentriMo [32] confirmed central enrichment of GC-rich motifs, validating the ChIP for both KLF1-ER and KLF1-E339K-ER proteins (Additional file 2: Figure S2). This altered specificity at the fifth nucleotide in the recognition sequence is consistent with the known mechanisms of DNA-contact of all SP/KLF zinc fingers (see below).

### KLF1-E339K binds to an altered motif in vitro

**Fig. 3:**
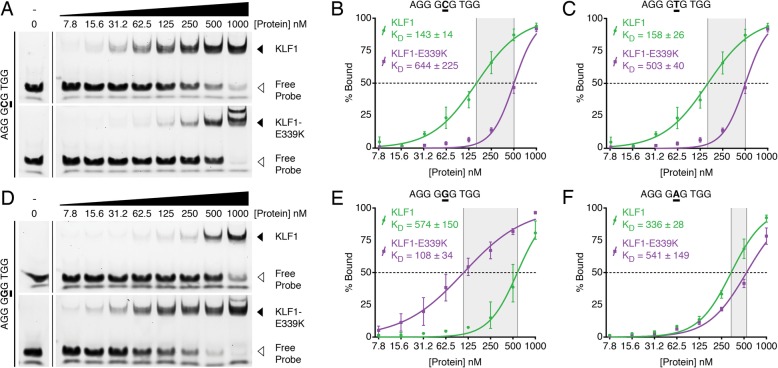
KLF1-E339K binds with strong affinity to an altered recognition sequence. **a** Saturation fluorescent gel shift assay of KLF1-zf and KLF1-E339K-zf (0–1000 nM) binding to a 2 nM probe representing the sequence at the *Alas2* enhancer binding site (AGG GCG TGG; C5). **b** Free versus bound probe were quantified and plotted from replicate gel shift assays to calculate the binding affinity constant (K_D_) of KLF1 (green) and KLF1-E339K (purple) to the *Alas2* enhancer binding site (AGG GCG TGG; C5). Curves were fit individually using with Hill Slope. Averaged K_D_ and its standard error are reported (*n* = 3). **c, e, f** Free versus bound probe were quantified and plotted from replicate (n = 3) gel shift assays as in A) with alternate probe sequences as indicated. **d** Saturation fluorescent gel shift assay of KLF1-zf and KLF1-E339K-zf (0–1000 nM) binding to a 2 nM probe representing an altered sequence at the *Alas2* enhancer binding site (AGG GGG TGG; G5)

To confirm aberrant binding was intrinsic to the variant KLF1-E339K-ER protein, we performed in vitro DNA-binding assays to compare the affinities of KLF1-zf and KLF1-E339K-zf (see Methods). We chose the *Alas2* erythroid gene enhancer, AGG GCG TGG, as the basis for these studies since it is a well-established direct KLF1 target gene [24]. We measured DNA-binding affinities for GST-purifiedKLF1-zf and KLF1-E339K-zf to probes for all four nucleotide variants at position 5 within an otherwise identical *Alas2*DNA-binding site. The binding affinity (K_D_) of KLF1-E339K-zf to the wild type probe (C5) was reduced almost 5-fold (K_D_ = 644 ± 225 nM) compared to KLF1-zf (K_D_ = 143 ± 14 nM) (Fig. 3a-b). Similarly, binding of KLF1-zf to a T5 variant (K_D_ = 158 ± 26 nM) was 3-fold higher than KLF1-E339K-zf (K_D_ = 503 ± 40 nM) (Fig. 3c). This is consistent with a previous publication using the β-globin promoter probe [33], since replacement of the C with a T as position 5 in the Alas2 enhancer site renders it identical to the normal β-globinpromoter-binding site. Interestingly, the affinity of KLF1-E339K-zf for a G5 variant (K_D_ = 108 ± 34 nM) is much greater (> 5-fold) than KLF1-zf (K_D_ = 574 ± 150 nM) (Fig. 3d-e). Lastly, neither protein bound strongly to the variant with a A at position 5 (Fig. 3f), but binding affinities of KLF1-zf and KLF1-E339K-zf for A5 variant were roughly equivalent (K_D_ = 350–500 nM). No binding to GST was observed. In summary, the biochemical binding data is consistent with the different in vivo preferences of KLF1-ER and KLF1-E339K-ER.

**Fig. 4:**
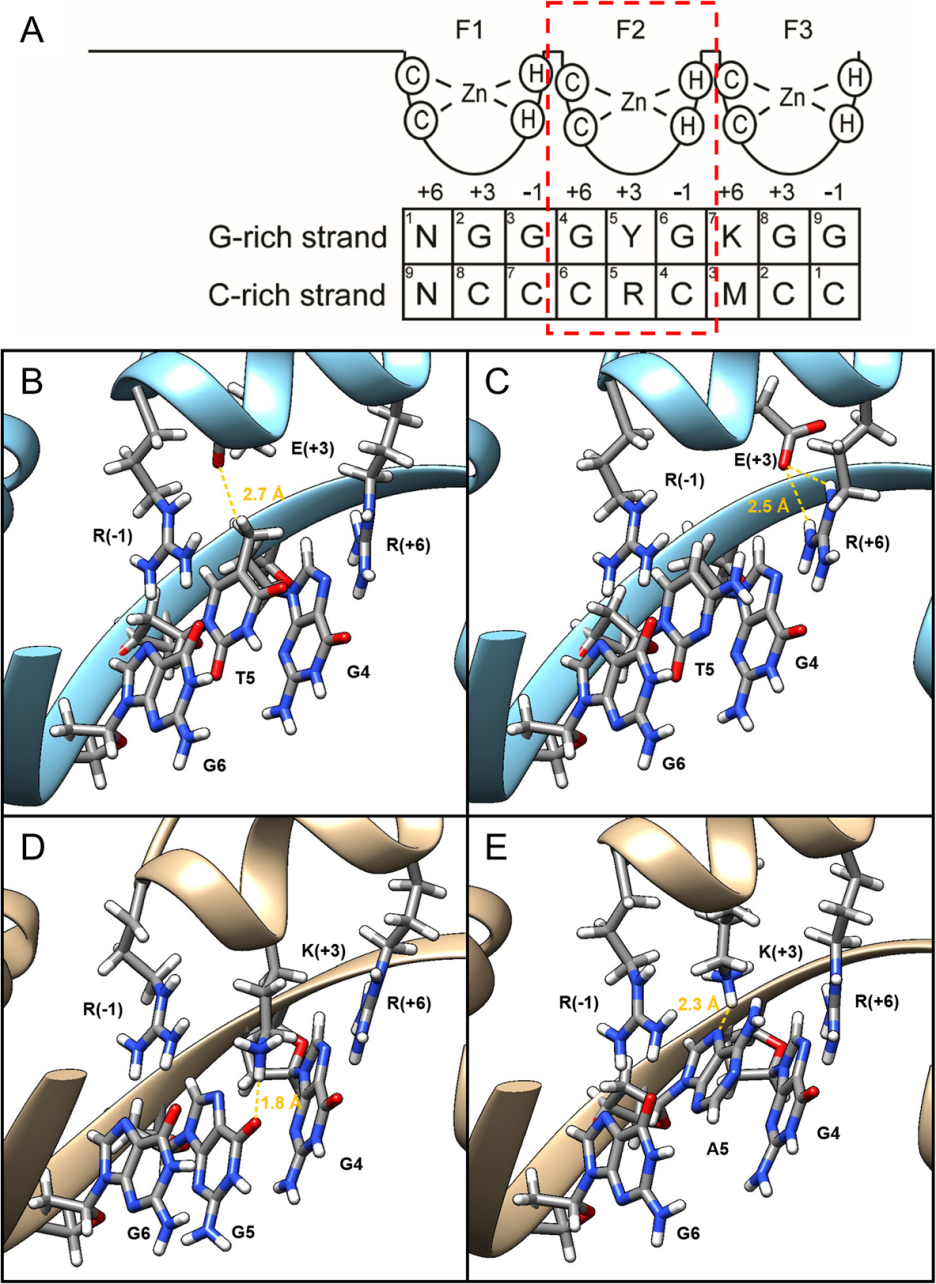
KLF1-E339K binds to the G-rich strand when purines are present at the fifth position, but lysine residue makes no contact with DNA. **a** Schematic of how KLF1 binds to CACCC-box motif via the G-rich strand. K = T/G, Y = T/C, M = A/C, R = A/G, N = any nucleotide. The panels in B-E focus on the interaction between the central triplet and ZF2. **b** Binding mode of the second zinc finger of KLF1 to a central GTG triplet on the G-rich strand, modeled on the crystal structure of KLF4 bound to the cognate GTG-containing DNA (PDB ID 5ke6). A weak C-H···O bond is the only contact between the glutamate (E) at the + 3 position and the central DNA base (T5). **c** Binding mode of the second zinc finger of KLF1 to a central GCG triplet on the G-rich strand, modeled on the crystal structure of KLF4 bound to the cognate GCG-containing DNA (PDB ID 2wbu). The glutamate does not directly contact the DNA but instead hydrogen bonds to the arginine (R) at the − 1 position. **d** Proposed binding mode of second zinc finger of KLF1-E339K to a central GGG triplet on the G-rich strand. The larger, positively charged lysine (K) sidechain extends towards the DNA and is able to act as a hydrogen bond donor, contacting the carbonyl group on G5 and forming a favorable hydrogen bond (shown in gold). **e** Proposed binding mode of second zinc finger of KLF1-E339K to a central GAG triplet on the G-rich strand. Substitution of guanine for adenine removes the hydrogen bond acceptor but an alternative, hydrogen bond is possible with the N7 atom of the adenine ring (shown in gold). This arrangement may also be possible when the central nucleotide is a G, however it is predicted that the N-H···O bond shown in D) is preferred, leading to a comparatively stronger affinity for GGG over GAG

Interpretation of these data at a molecular level is speculative in the absence of structural data on KLF1. However, the residues of finger 2 which contact the central DNA triplet in the homologous KLF4 are entirely conserved in KLF1 (Fig. 4a), meaning that some level of insight can be gleaned from published structures of KLF4 bound to different DNA sequences [19, 34, 35]. In KLF4, recognition of the central T involves a relatively weak C-H···O interaction between the T5 methyl group and the glutamic acid [35] . When the central base is a C, there is no contact with the glutamic acid; rather the glutamic acid side chain appears to stabilize the position of the nearby arginine which contacts the G at position 4 [34]. Again, these residues are 100% conserved between KLF1 and KLF4 and so the mechanism of recognition is also expected to be conserved (Fig. 4b-c).

Modeling the E325K mutation in KLF1 based on these KLF4 structures provides a plausible explanation for the change in preference from a pyrimidine to a purine at the central base, and in particular the enhanced binding to sequences containing a central GGG triplet. The larger sidechain of the lysine residue is long enough to form what is predicted to be a strong N-H···O hydrogen bond with the carbonyl group of the guanine ring (Fig. 4d). According to this model, adenine, which does not have a carbonyl group, would not be able to interact in this same way with the lysine sidechain, but may still contact it via a possibly weaker N-H···N hydrogen bond formed with the N7 nitrogen (Fig. 4e).

### Change of DNA binding specificity leads to dysregulation of gene expression

**Fig. 5:**
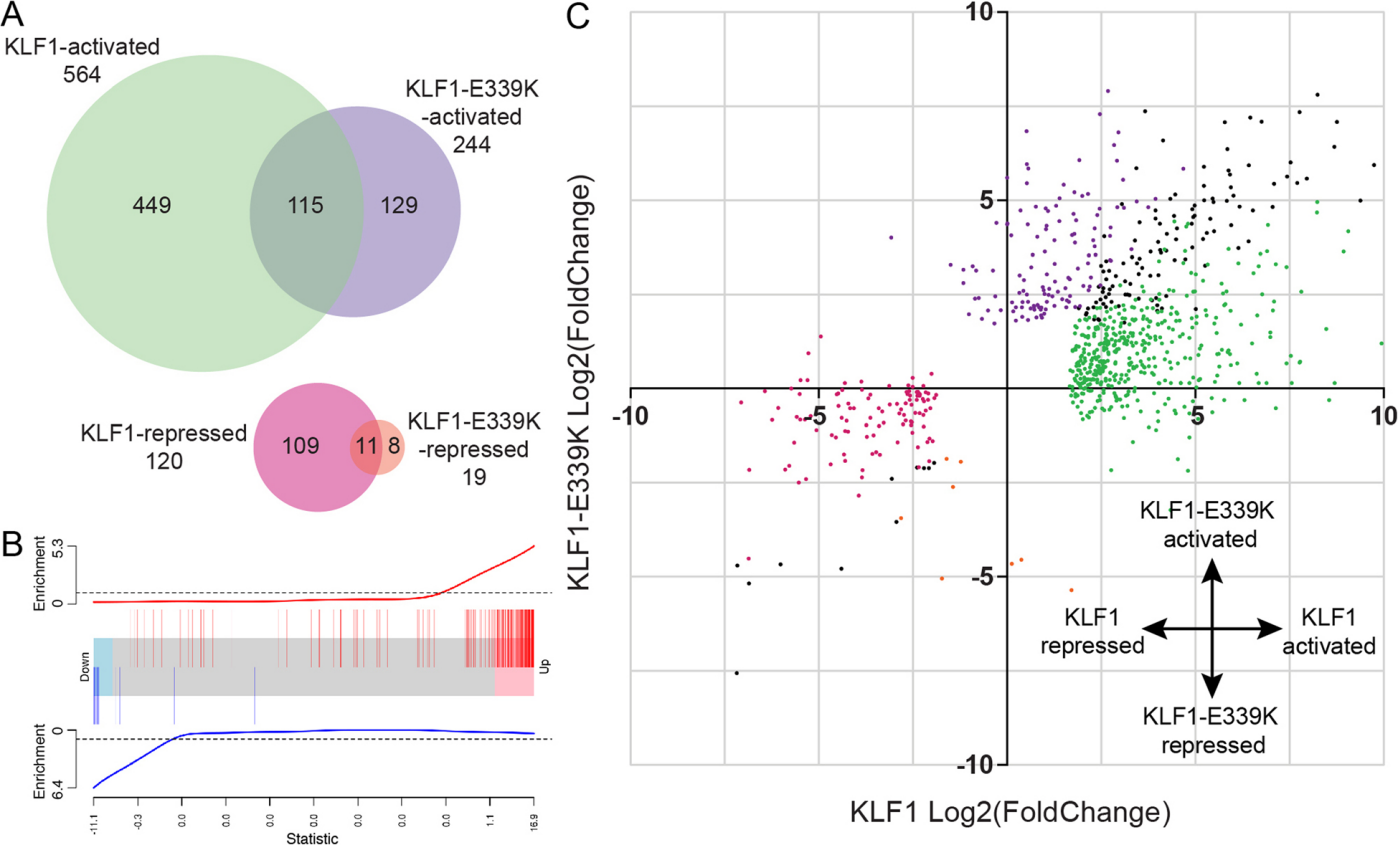
KLF1-E339K-ER causes dysregulation of gene expression via aberrant activation of genes. **a** Venn diagram showing the overlap of differentially expressed genes (DEGs) from K1-ER and K1-E339K-ER 4sU-RNA-seq analysis. **b** Barcode plot demonstrating correlation of differentially expressed genes due to KLF1-E339K-ER (comparison of K1-E339K-ER cells versus K1; FDR < 0.05, Additional file 6: Table S2; red bars: upregulated genes; blue bars: downregulated genes) relative to gene expression changes due to KLF1-ER. Horizontal axis shows moderated t-statistic values for KLF1-ER. Red and blue worms show relative enrichment of up and downregulated genes (ROAST *P*-value < 0.01 for upregulated genes). **c** Scatter-plot of the log_2_ (fold change) of all genes called as significant in either K1-ER or K1-E339K-ER 4sU-RNA-seq analysis. Colors are consistent with those in the Venn diagram (A). Black dots represent genes that are significantly differentially regulated by both TFs

To assess the transcriptional response to aberrant TF binding, we performed RNA-seq analysis on newly-transcribed RNA by utilizing 4-Thiouridine (4sU)-labeling [36, 37]. This has the advantage of enriching for immediate and direct transcriptional events following DNA binding. Three clonally independent lines of K1 and KLF1-E339K-ER cells were induced with 200 nM 4-OHT (or ethanol control) and treated with 500 mM 4sU for 1 h. 4sU-labeled RNA was isolated as previously described [24, 36]. Enrichment for primary transcripts was validated by RT-PCR (Additional file 3: Figure S3). Differentially expressed genes (DEGs) were analyzed using the R package: Limma-voom [38] (see Methods). Following normalization and linear modelling of the data, a total of 244 genes were significantly up-regulated, and 19 genes were significantly down-regulated in response to KLF1-E339K-ER using a minimum log-fold-change of 2 (Additional file 6: Table S2). This was compared to our KLF1-ER target genes determined by comparison of K1-ER and K1 cell lines using the same analysis parameters (Fig. 5a) [24]. A total of 564 genes were significantly upregulated, and 120 genes were significantly down-regulated in response to KLF1 induction with 126 of these genes differentially expressed in both datasets.

**Fig. 6:**
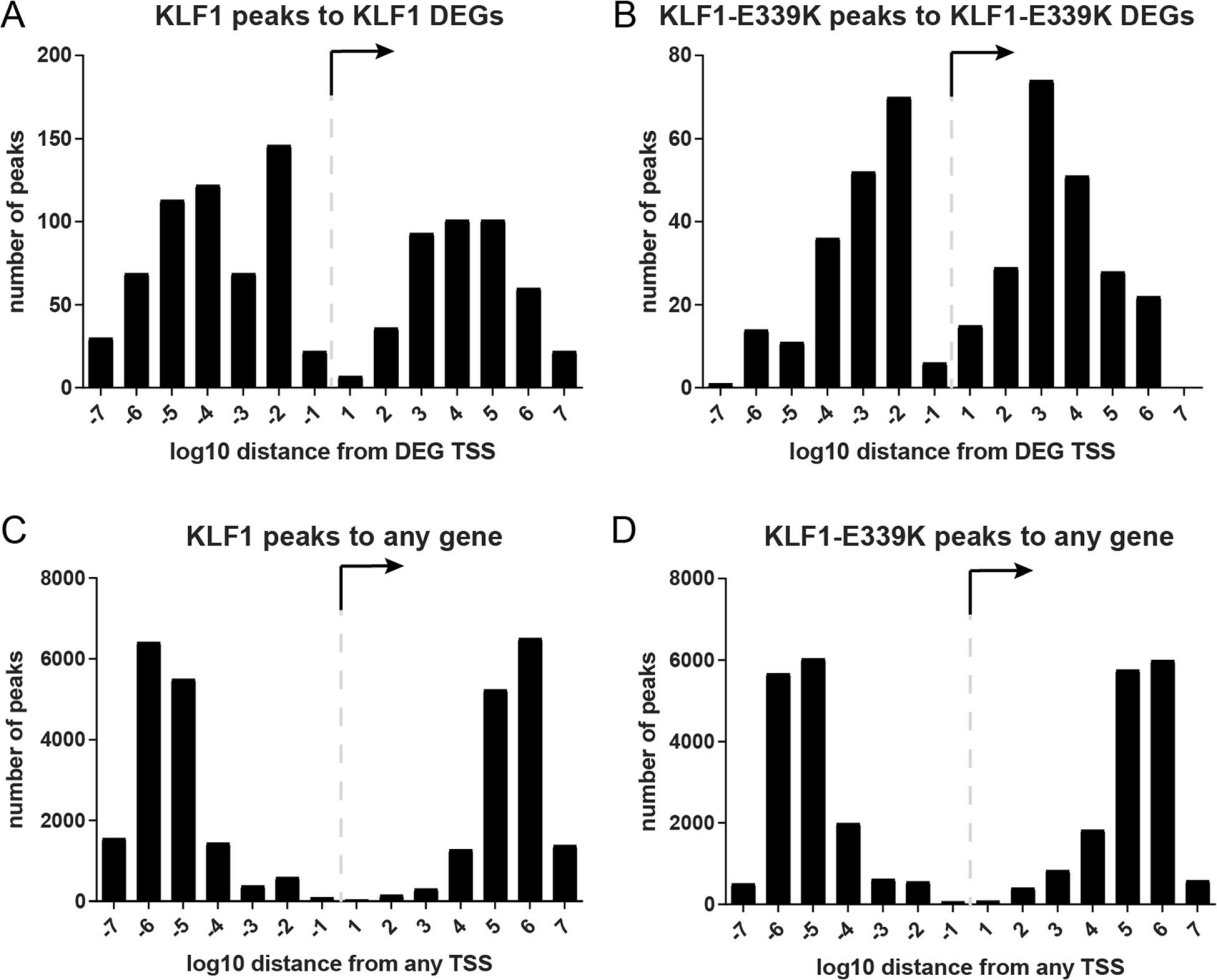
Aberrant transcription is a direct result of KLF1-E339K-ER binding. **a** Graph of the number of KLF1-ER ChIP-peaks versus distance (log_10_ nt) and direction from the TSS of K1-ER DEGs.**b** Graph of the number of KLF1-ER ChIP-peaks versus distance (log_10_ nt) and direction from the TSS of all genes within the genome. **c** Graph of the number of KLF-E339K-ER versus distance (log_10_ nt) and direction from the TSS of K1-E339K-ER DEGs. **d** Graph of the number of KLF1-E339K-ER peaks versus distance (log_10_ nt) and direction from the TSS of all genes within the genome

We then used ROAST [39] to compare these gene signatures and found significant enrichment of genes induced by KLF1-E339K-ER in K1-ER cells (*P* < 0.01) (Fig. 5b). Direct comparison of the DEG log2-fold-change by both factors highlights they are primarily transcriptional activators and suggests a hypomorphic function for KLF1-E339K-ER (Fig. 5c).To confirm that ectopic transcription (Fig. 5c, purple data points) is a consequence of TF binding, we surveyed the distance to the closest KLF1-ER or KLF1-E339K-ERChIP-seq peak to the nearest TSS of DEGs in K1-ER (Fig. 6a) or K1-E339K-ER (Fig. 6c) RNA-seq, respectively. As a control, the distance to the closest KLF-ER 1 or KLF1-E339K-ERChIP-seq peak for any TSS in the genome was also calculated (Fig. 6b-d). ChIP-seq peaks were markedly enriched within 10 kb of the TSS of DEGs for both KLF1 and KLF1-E339K compared to the TSS of non-regulated genes indicating that both KLF1 and KLF1-E339K directly activate local gene expression.

Finally to explore the pathways that are affected by ectopic expression we analyzed the KLF1-E339K-ER DEGs using a gene set analysis toolkit (webgestalt) [40]. Interestingly, gene ontology (GO) analysis of uniquely KLF1-E339K-ER activated genes revealed no enrichment for erythroid processes or pathways (KEGG), supporting the notion that KLF1-E339K-ER aberrantly activates genes randomly throughout the genome (Additional file 4: Figure S4). The top 5 significant pathways identified by this analysis were: Aldosterone-regulated sodium reabsorption (renin-angiotensin-aldosterone system in the adrenal cortex), EGFR tyrosine kinase inhibitor resistance (acquisition of resistance in non-small-cell lung cancer), TGF-beta signaling pathway (cell growth, differentiation and apoptosis), Relaxin signaling pathway (hormone released in the birth canal before delivery) and colorectal cancer. These pathways are functionally distinct to each other, are involved in separate tissues, and in combination, likely lead to the cell death observed in K1-E339K-ER cells due to derailment of the normal erythroid transcriptional program.

### KLF1-E339K-ER and KLF1-E339D-ER exhibit distinct binding specificity and target genes

**Fig. 7:**
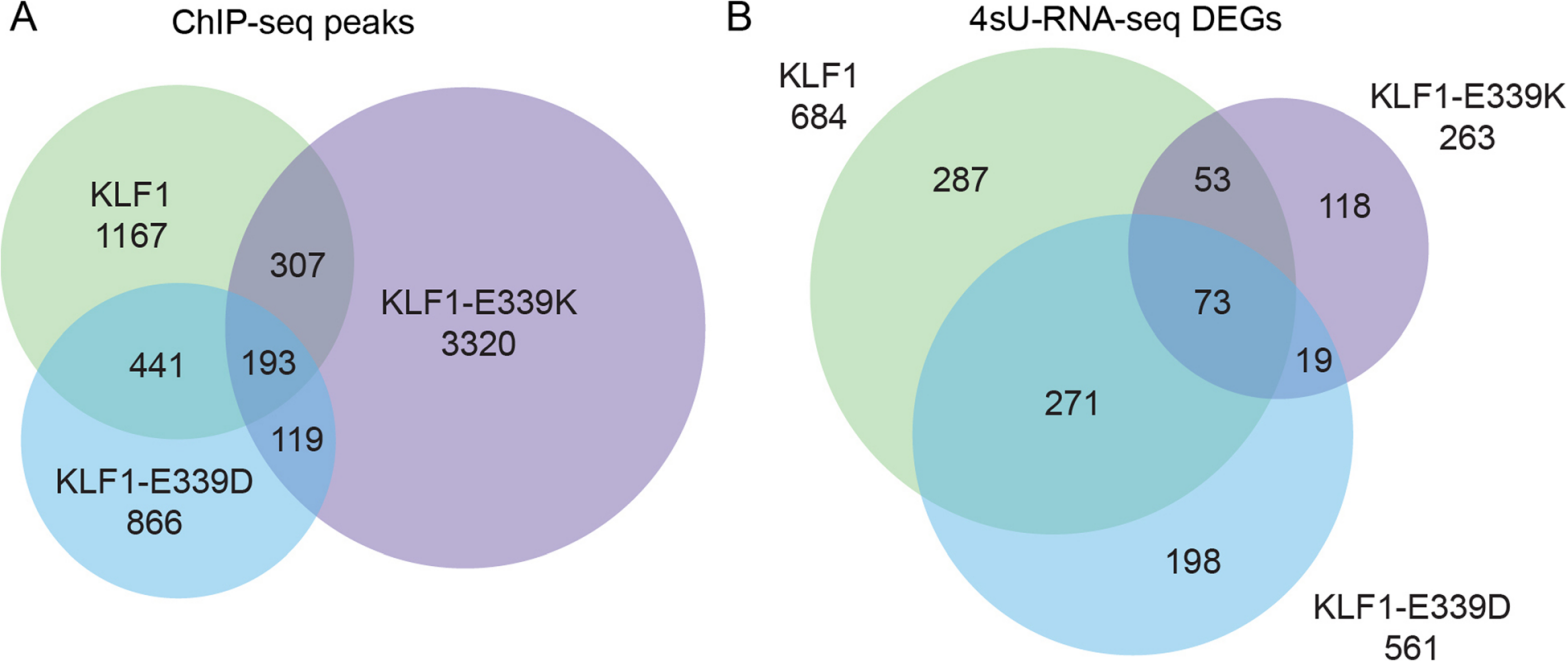
KLF1-E339K-ER and KLF1-E339D-ER bind different sites in the genome to regulate different sets of genes. **a** Proportional Venn diagram of ChIP-seq peak overlaps between wildtype KLF1-ER, KLF1-E339K-ER and KLF1-E339D-ER. Peaks are considered shared if the peak summits are within 250 bp of each other. **b** Proportional Venn diagram of DEGS in response to wildtype KLF1-ER, KLF1-E339K-ER or KLF1-E339D-ER induction

Although both humans with CDA IV and *Nan* mice display dominant hemolysis, the change in DNA-binding specificity for the E > K and E > D variants [24] in KLF1-ER are very different. To evaluate this further, we determined the overlap of peaks from KLF1-ER, KLF1-E339K-ER and KLF1-E339D-ER [24] ChIP-seq data sets using DiffBind (Fig. 7a). Only 193 from > 7000 combined sites are occupied by all three versions of KLF1-ER. There is also minimal overlap between KLF1-E339D-ER and KLF1-E339K-ER -occupied sites (312 of > 5000 total sites) consistent with the different binding specificities of the two proteins.

We also determined whether there was any differential gene expression in the same cell system between the three groups (Fig. 7b). Once again, there was little overlap between either activated or the repressed gene sets for each version of KLF1-ER. This strongly suggests the function of the two mutant versions of KLF1 are distinct. Thus, we suggest the similar biological consequence of hemolysis is most likely due to the combination of expression of many genes and proteins which together add up to a toxic mixture rather than a few specific ones.

## Discussion

CDA IV (OMIM: 613673) is a dominantly inherited anemia caused by a point mutation in the second zinc finger of KLF1 (c.973G > A; p.E325K) [16]. The dominant nature of this mutation sets it apart from all other human *KLF1* mutations described to date [3], but it is similar to the dominant murine *Nan* mutation in many respects [20, 25]. Previous work did not explain the dominant phenotype but has shown the E > K variant leads to reduced DNA binding to the β-globin promoter [33]. We confirmed this result but also showed for the first time the E > K (CDA IV) mutation results in an altered DNA-binding specificity. This results in less binding at important canonical target sites such as the *β-glob*in gene promoter, *Alas2* and *E2f2* enhancers, but also aberrant binding to new sites resulting in transcriptional activation of non-erythroid genes. Thus, the CDA mutation (KLF1-E339K) is similar to the *Nan* mutation (KLF1-E339D), exhibiting both a hypomorphic and neomorphic function.

Nucleotide analysis of in vivo occupied sites and in vitro affinity assays revealed KLF1-E339K prefers sequences containing a guanine at the central position on the G-rich strand rather than a pyrimidine (T or C). This nucleotide is directly contacted by + 3 amino acid in ZF2, which is highly conserved across all KLF and SP family members. The substitution of a polar (negative) sidechain for a larger, polar (positive) sidechain appears to result in a switched nucleotide preference at the central position from a pyrimidine to guanine. This is consistent with our understanding of the physical interactions between the + 3 amino acid in ZF2 of KLF/SP proteins and the DNA motif they are known to recognize [19, 34].

This work highlights the importance of the use of genomic techniques such as ChIP-seq to understand complex diseases caused by missense mutations in DNA-binding domains of TFs. We suggest this scenario is far more common than currently appreciated and not limited to TFs of the zinc finger class. There are reports of missense mutations in other DNA-contacting amino acids in ZF1 of KLF1 [3]. In fact, the A298P mutation (at + 6 in finger 1) is quite common in certain populations [10, 41]. Heterozygotes for this mutation have a mild phenotype but compound heterozygotes (with a co-inherited null allele) have severe hemolysis [10]. It is reasonable to conclude that the A289P mutation might also have a neomorphic DNA-binding function with transcriptome consequences. We predict a change in DNA-binding specificity for A298P in ZF1 which is different again from both E339K and E339D mutations in ZF2. Interestingly, there is also a dominant mutation in murine *Klf3* at the + 3 position in ZF1 (H275R) which was discovered in an ENU-generated mouse with congenital heart disease (CHD) [42]. Since *Klf3*^+/−^ mice have no overt CHD, we suggest the H275R mutation is also likely to lead to aberrant DNA-binding and transcriptome consequences that are toxic to muscle or endothelial cells in the heart.

Based on our combined in vivo and in vitro data, it is likely that CDA IV is caused by a general dysregulation of gene expression in developing erythrocytes that derails differentiation and induces hemolysis. While KLF1-E325K is likely to be hypomorphic with respect to some known KLF1 target genes such as CD44 and ICAM4, this study highlights that it is also likely to be neomorphic with respect to additional non-erythroid genes. In this study have employed a murine system due to the lack of available *KLF1*^−/−^ human erythroid cell lines, so the precise identity of dysregulated genes in human CDA IV cells cannot be discovered. However, we propose the observed neomorphic functionalization of KLF1-E339K underpins the molecular mechanism of disease in CDA IV.

## Conclusions

This work provides insight into the dominant molecular mechanism of Congenital Dyserythropoietic Anemia Type IV (CDA IV) which is caused by a point mutation (E325K) in the DNA-binding domain of KLF1. We found this mutation alters the DNA binding specificity KLF1 and this leads to aberrant binding in vivo at a few thousand sites scattered across the genome. While many of these binding events have no consequence, a significant number (~ 5%) lead to dysregulation of nearby gene expression. We show that the effects of this mutation are distinct from those observed from mutation of an equivalent residue in mice (E339D), which is consistent with the different in vitro DNA-binding specificities of the two variants. Since all KLF/SP family proteins bind DNA in an identical fashion, and many other more distantly related C2H2 transcription factors bind DNA in a similar fashion, these results may be generally applicable to mutations in all family members. Importantly, they explain how certain mutations in the DNA-binding domain of sequence specific transcription factors can generate neomorphic functions that result in autosomal dominant disease.

## Methods

### Generation of cell lines

K1 cells, previously known as B1 cells, were previously generated in our laboratory from *Klf1*^−/−^ fetal liver cells [27]. The KLF1-E339K open reading frame was cloned in frame with ERα into MSCV-IRES-GFP, generating the plasmid MSCV-KLF1-E339K-IRES-GFP. The plasmid was transfected into GP + E86 cells to generate a stable retrovirus producing clone. K1 cells were infected and sorted for GFP+ by FACS. K1-ER cells which express wild type KLF1-ER were generated in the same way [24]. 4-OHT induced nuclear translocation of the transgenes was confirmed by western blotting using a mouse monoclonal antibody raised against ERα (ThermoScientific).

### Chromatin immunoprecipitation and sequencing (ChIP-seq)

K1-E339K-ER cell lines were incubated in 200 nM 4-OHT (or ethanol vehicle control) for 3 h prior to crosslinking with 0.4% formaldehyde. KLF1-E339K-ER ChIP was performed using a mouse monoclonal antibody against ERα (ThermoScientific, ss-315-P) 3 hours post-induction with 4-OHT as previously described [24]. Enrichment of specific target sites in ChIPed DNA was validated by qPCR. Samples were pooled and used to generate Ion Xpress™ Plus fragment libraries and sequenced on the Ion Proton platform. Reads were mapped to the mouse genome (mm9) using TMAP [43]; duplicate reads and multi-mapped reads were excluded. Peaks were called using MACS2 [29] and GEM [30]. We used EaSeq to plot read densities of ChIP-seq data [44] and DiffBind [31] to analyze to examine differential enrichment between peaksets.

### 4sU-RNA isolation and sequencing (4sU-RNA-seq)

4sU-RNA-seq was performed as previously described [24]. Three clonally independent lines of K1 and K1-E339K-ER cells were incubated with 200 nM 4-OHT (or ethanol control) and 500 mM 4sU for 1 h. 4sU-labeled RNA was isolated as previously described [24, 36]. Enrichment for primary transcripts was validated by RT-PCR.4sU-labeled RNA was used to generate Ion Xpress™ Plus fragment libraries and sequenced on the Ion Proton platform. Reads were mapped to the mouse genome (mm9) using Tophat2 [45] and TMAP [43]. Reads were further filtered to remove reads with low mapping quality (> 30). Significantly differentially regulated genes (DEGs) were determined using the R package: Limma-Voom [38].

### Recombinant protein purification

KLF1-E339K zinc fingers together with the nuclear localization signal (residues 261–376) were cloned into pGEX-6P1 using *BamHI* and *EcoRI* restriction enzymes, after amplification from MSCV-CDA-KLF1-IRES-GFP (see above). GST-KLF1-zf and GST-KLF1-E339K-zf proteins were expressed and purified as previously described [24] with minor deviations. Rosetta (DE3) *E. coli* transformed with plasmids were induced with 0.4 mM isopropyl β-D-1-thiogalactopyranoside (IPTG) and 1 μM ZnCl_2_ (to assist in protein folding) then maintained at 37 °C for 3 h before collection. Cell pellets were resuspended in cold lysis buffer (50 mM Tris-HCl pH 7.4, 150 mM NaCl, 5 mM EDTA, 50 μM ZnCl_2,_) and lysed with lysozyme in 10% NP-40, 1 mM DTT, 0.2 mM PMSF and cθmplete protease inhibitor cocktail (Roche). Cell lysates were sonicated and the insoluble fraction was pelleted and discarded. GST-KLF1-zf protein was purified using glutathione-sepharose beads (GE Healthcare) and non-specifically bound protein was removed with a wash buffer (50 mM Tris-HCl pH 7.4, 500 mM NaCl, 100 μM ZnCl_2_, 10% NP-40, 0.2 mM PMSF and cθmplete protease inhibitor cocktail). Recombinant protein purity and quantity was determined by Coomassie-stainedSDS-PAGE with protein size standards of known quantity.

### EMSA

Electromobility shift assays were performed as previously described with some modification [46]. Binding of GST-KLF1-zf and GST-KLF1-E339K-zf were tested against 2 nM FAM labelled probes representing the binding site of the *Alas2* gene enhancer region (3′-GAGCCC**AGGGCGTGG**GAGAGA-FAM) and the same probe with variants at the fifth position of the CACC motif (3′-GAGCCC**AGGGAGTGG**GAGAGA-FAM, 3′-GAGCCC**AGGGTGTGG**GAGAGA-FAM, 3′-GAGCCC**AGGGGGTGG**GAGAGA-FAM). Gels were scanned on a Typhoon Trio Variable Mode Imager System (GE Healthcare) and band density (free and bound) was determined by ImageQuant (GE Healthcare). To calculate the binding affinity constant (K_D_), the [probe] was kept constant and the [protein] was altered in different reactions. The [protein] at which 50% is bound represents the K_D_. Curves were fit individually using Specific Binding with Hill Slope. Averaged KD and its standard error are reported.

### Software

Statistics analysis was performed with GraphPad Prism version 6.04 for Windows, GraphPad Software, La Jolla California USA, www.graphpad.com. Alignments of human and mouse KLF1 proteins was performed using the program Geneious version R7 [47]. Density plots of aligned reads were generated using EaSeq [44], available from: http://easeq.net. Proportional Venn Diagrams were produced using EulerAPE [48]. Modeling of protein-DNA structures was carried out in UCSF ChimeraX [49]. Structures of the second zinc finger of KLF4 bound to the central DNA triplet of the G-rich strand were modelled initially based on structures of KLF1 bound to DNA [34, 35]. Mutations were introduced and structures then optimized using the *swapaa*, *tug* and *minimize* functions within the OpenMM dynamics environment. Structural images were then prepared in ChimeraX.

## Additional files

Additional file 1: **Figure S1**. Inducible cell lines to study human CDA type IV. Full length western blot of nuclear extracts from cell lines generated in this study as shown in Fig. 1b. The blot shows presence of KLF1-ER in the nucleus after induction of 4-OHT (+) in a K1-ER cell line and 3 independent clones of the K1-E339K-ER cell line. (JPG 645 kb)

Additional file 2: **Figure S2**. Central enrichment of identified PWM. CentriMo analysis of PWM (motif) identified by MEME is found closest to the summit of ChIP-seq peaks for KLF1-E339K-ER (A) and KLF1-ER (B). (JPG 302 kb)

Additional file 3: **Figure S3**. 4sU-labeled RNA Enrichment. Validation by qRT-PCR of 4sU-RNA isolation from total RNA. Samples used for RNA-seq analysis were validated for enrichment of primary transcript (4sU-labeled) relative to mature *Hprt* (A) and *E2f2* (B) transcripts. (JPG 305 kb)

Additional file 4: **Figure S4**. Gene Set Analysis of KLF1-E339K-ER DEGs. Gene signatures from the analysis of K1-E339K-ER cells versus K1 were tested using the gene set analysis toolkit on the KEGG pathway using Entrez IDs (Additional file 6: Table S2). Significant enrichment (FDR < 0.05) of a number of distinct is pathways shown with enrichment ratio. (JPG 250 kb)

Additional file 5: **Table S1**. Comparison of KLF1-E339K-ER and KLF1-ERChIP-seq peaks. Annotated list of all consensus peaks from differential binding analysis. Closest TSS to each peak is called as well as closest up- and down-regulated gene from RNA-seq analysis. (XLSX 481 kb)

Additional file 6: **Table S2**. K1-E339K-ER4sU-RNA-seq DEGs. Gene expression changes following activation of KLF1-E339K-ER compared to K1 (*Klf1*^*−/−*^) cells. Differentially Expressed Genes (DEGs) using a false discovery rate (FDR) of 0.05 with the treat method employing a minimum log-fold-change of 2. (XLSX 1979 kb)

## Data Availability

The datasets generated and/or analyzed during the current study are available in the Gene Expression Omnibus (GEO) under the identifiers GSE92620 [https://www.ncbi.nlm.nih.gov/geo/query/acc.cgi?acc=GSE92620], GSE98801 [https://www.ncbi.nlm.nih.gov/geo/query/acc.cgi?acc=GSE98801] and GSE71396 [https://www.ncbi.nlm.nih.gov/geo/query/acc.cgi?acc=GSE71396].

## Abbreviations

4-OHT: 4-hydroxytamoxifen
4sU: 4-Thiouridine
CDA IV: Congenital Dyserythropoietic Anemia type IV
CHD: congenital heart disease
DEG: Differentially expressed gene
E: Embryonic day
EMSA: Electromobility shift assay
ER: Estrogen Receptor
ER™: tamoxifen-inducible estrogen receptor,
FBE: full blood examination
GO: gene ontology
GWAS: Genome-wide association study
K_D_: binding affinity
KLF1: Krüppel-like factor 1,
MCV: mean corpuscular volume
*Nan*: neonatal anemia mouse model
PWM: Position weighted matrix
TF: transcription factor
zf: zinc finger domain
ZF2: second zinc finger
