# Supplementary figures and images for: Corrupted DNA-binding specificity and ectopic transcription underpin dominant neomorphic mutations in KLF/SP transcription factors
Source: BMC Genomics. 2019 May 24;20:417. doi: 10.1186/s12864-019-5805-z (PMC6534859; doi:10.1186/s12864-019-5805-z)

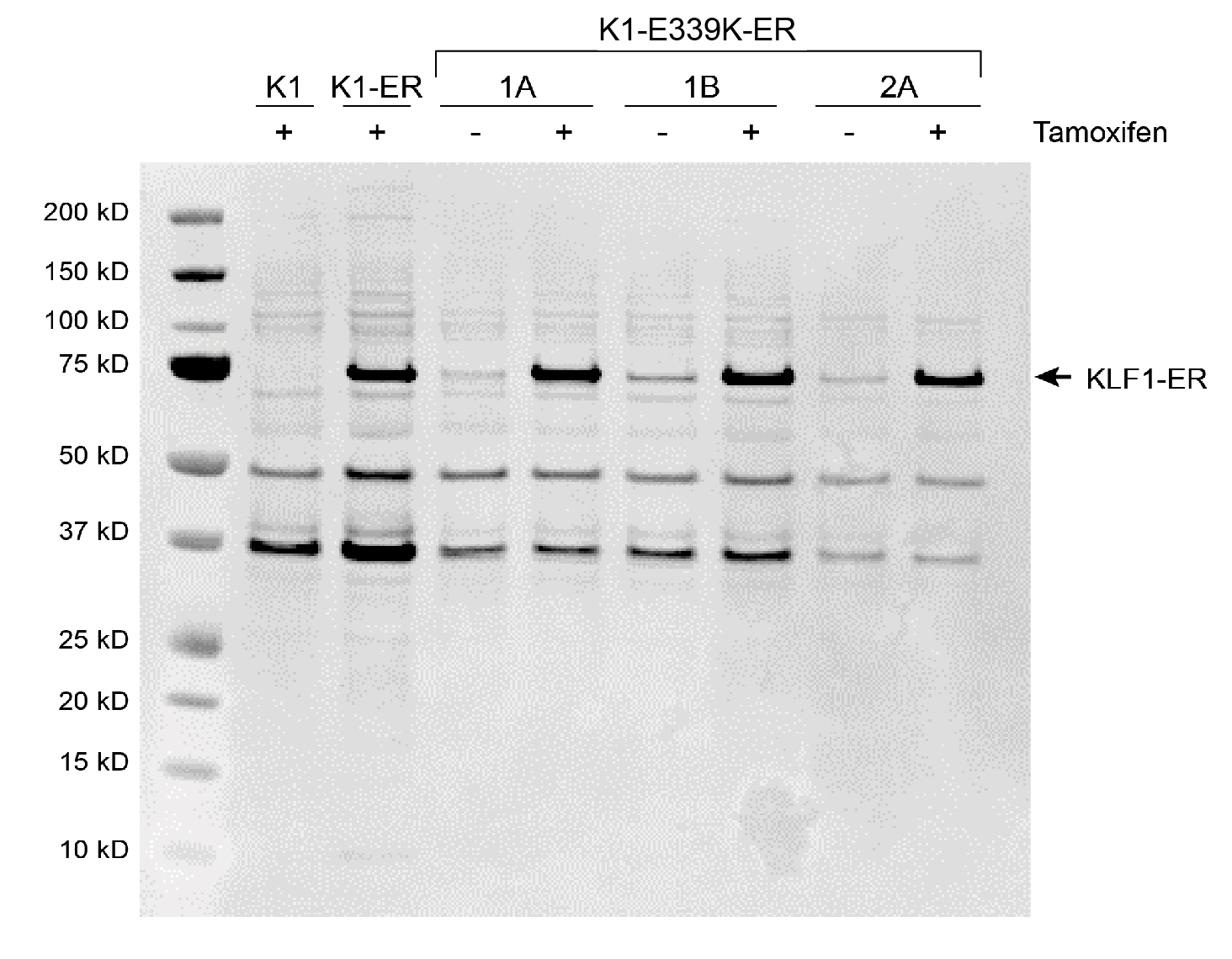

Supplement: Supplementary file 1 — Figure S1. Inducible cell lines to study human CDA type IV. Full length western blot of nuclear extracts from cell lines generated in this study as shown in Fig. 1b. The blot shows presence of KLF1-ER in the nucleus after induction of 4-OHT (+) in a K1-ER cell line and 3 independent clones of the K1-E339K-ER cell line. (JPG 645 kb) [file 12864_2019_5805_MOESM1_ESM.jpg]

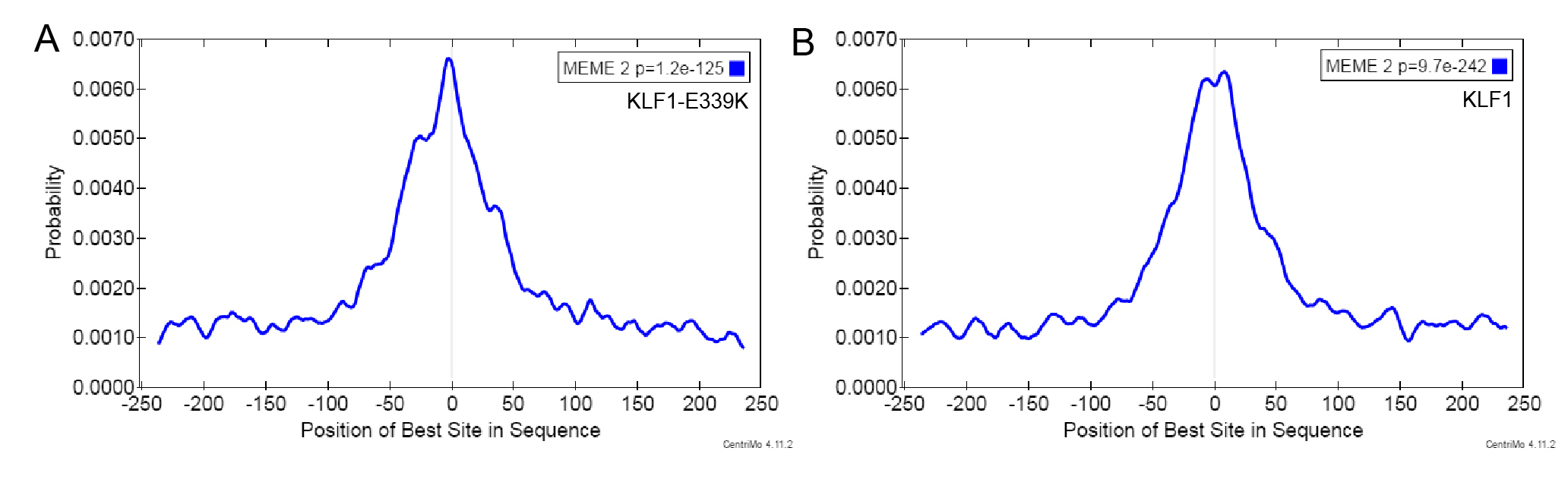

Supplement: Supplementary file 2 — Figure S2. Central enrichment of identified PWM. CentriMo analysis of PWM (motif) identified by MEME is found closest to the summit of ChIP-seq peaks for KLF1-E339K-ER (A) and KLF1-ER (B). (JPG 302 kb) [file 12864_2019_5805_MOESM2_ESM.jpg]

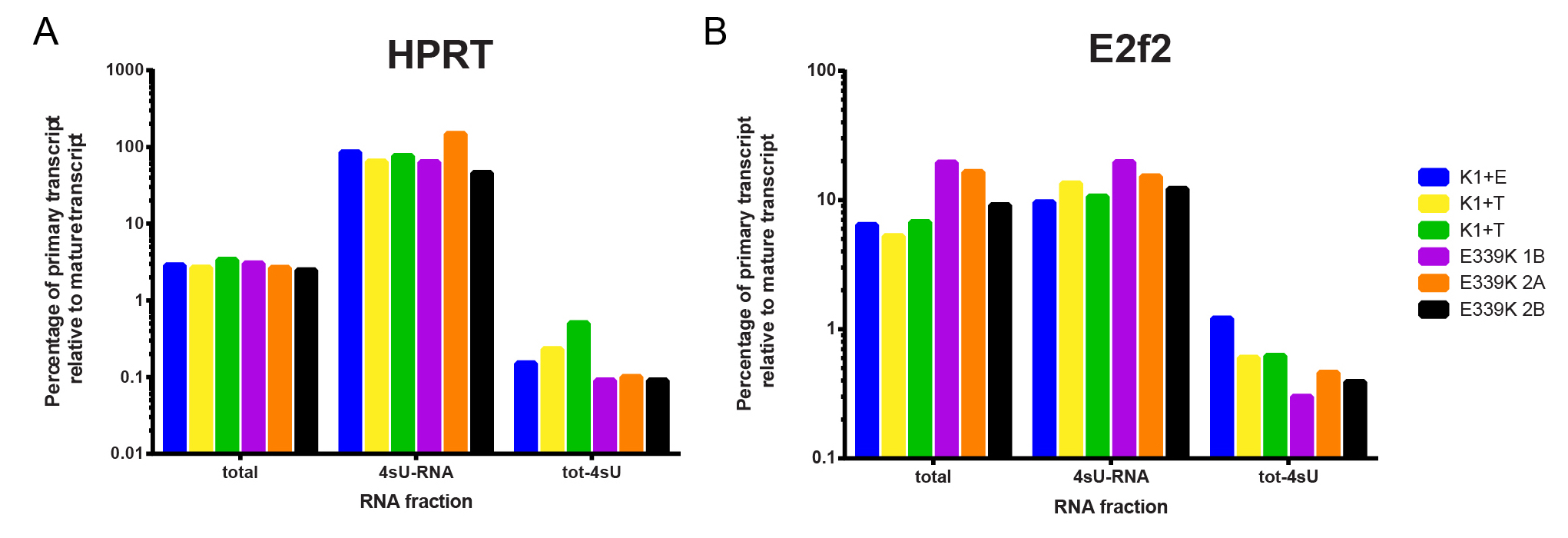

Supplement: Supplementary file 3 — Figure S3. 4sU-labeled RNA Enrichment. Validation by qRT-PCR of 4sU-RNA isolation from total RNA. Samples used for RNA-seq analysis were validated for enrichment of primary transcript (4sU-labeled) relative to mature Hprt (A) and E2f2 (B) transcripts. (JPG 305 kb) [file 12864_2019_5805_MOESM3_ESM.jpg]

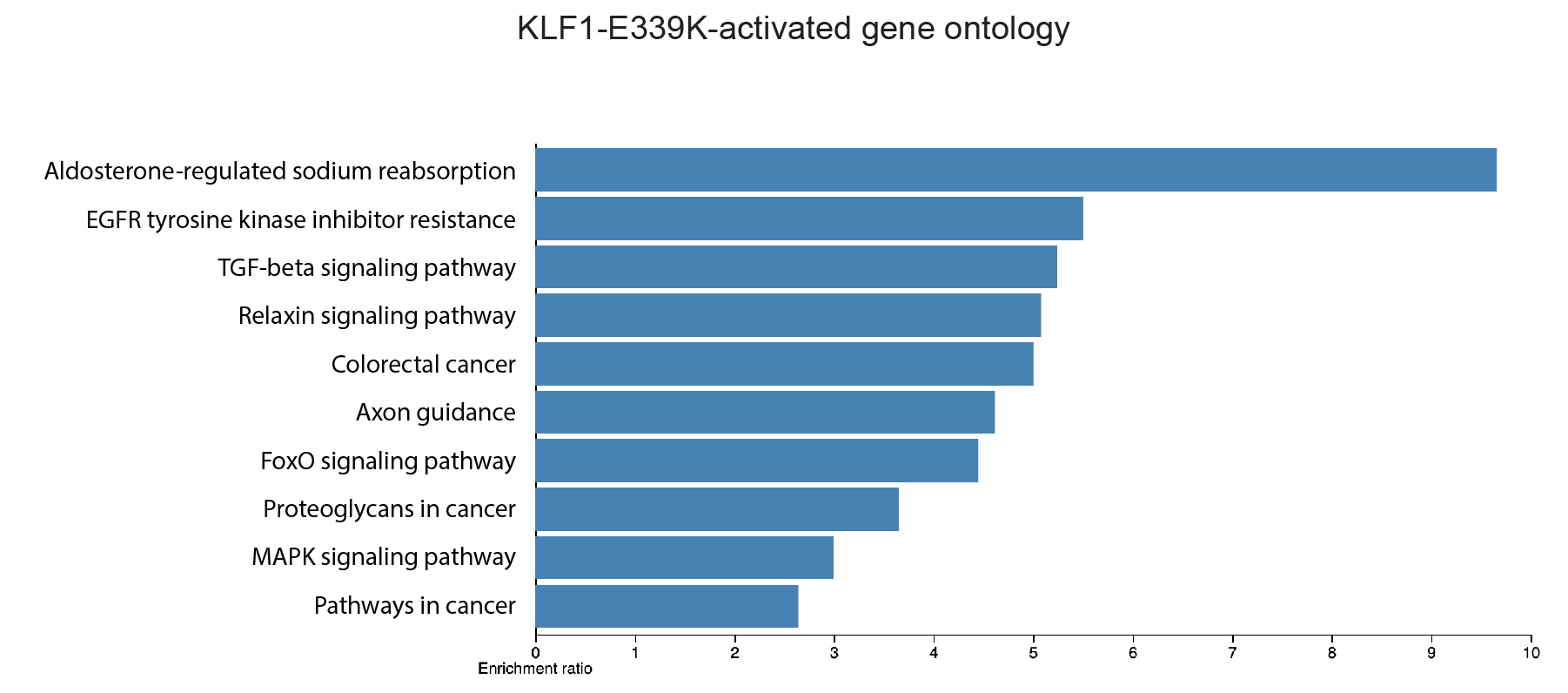

Supplement: Supplementary file 4 — Figure S4. Gene Set Analysis of KLF1-E339K-ER DEGs. Gene signatures from the analysis of K1-E339K-ER cells versus K1 were tested using the gene set analysis toolkit on the KEGG pathway using Entrez IDs (Additional file 6: Table S2). Significant enrichment (FDR < 0.05) of a number of distinct is pathways shown with enrichment ratio. (JPG 250 kb) [file 12864_2019_5805_MOESM4_ESM.jpg]
